# Leadership in health and medical education: lessons from a symposium on health sector development in Ghana

**DOI:** 10.4314/gmj.v57i1.11

**Published:** 2023-01

**Authors:** Cephas K Avoka, Martha S Nabila, Akua Addy, Abena Okoh

**Affiliations:** 1 Cohort 16, Faculty of Public Health, Ghana College of Physicians and Surgeons, Accra, Ghana

**Keywords:** leadership, medical education, health sector development, health symposium, mentorship

## Abstract

The Ghana College of Physicians and Surgeons (GCPS) has established an annual leadership symposium celebrating innovative leadership in the health sector. The 2022 symposium under the theme “Health Sector Development in Ghana; The Power of Good Leadership” was held in honour of Professor Samuel Ofosu-Amaah (the laureate), an Emeritus Professor of Public Health at the University of Ghana, about his leadership legacy. This article reflects on the leadership challenges in the health sector, the lessons learnt from the symposium, and the way forward.

Leadership challenges identified in the health sector included the need for mentorship and coaching, the importance of teamwork and networking for delivering high-quality healthcare, and the role of leadership and governance in the health system. Key lessons from the symposium focused on skills in leading an event organisation, effective collaboration and teamwork, and learning from recognising prominent leaders' contributions to the health sector while these leaders are still alive. Key lessons from the personal and professional life of the laureate included a focus on giving back to the community, building mentorship of health leaders, being a catalyst of change, leadership and governance in public health institutions and publication of research findings.

Suggestions were made to name the School of Public Health of the University of Ghana after Professor Ofosu-Amaah, to include a leadership and management module in all training modules at the GCPS and to establish a health leadership “Observatory” to focus on research on how leadership influences relevant health sector policy issues.

**Funding:**

The World Health Organization (WHO) country office in Ghana funded the symposium.

## Introduction

Good leadership is critical in Ghana's drive towards achieving the Sustainable Development Goals.^1^ Good leadership ensures that prudent policies are created and drives the implementation of such policies. These policies thus create the platform for service programs, protocols, and procedures that ensure equity in delivering health services nationwide. This kind of leadership must come from all societal sectors, including the government, private sector, groups, and individuals.^2^ As the country advances toward attaining Universal Health Coverage, there is a need for health professionals to exhibit good leadership competencies needed to drive this reform. Thus, good leadership, a highly valued component of healthcare education, is needed in training health sector professionals to enable them to apply the principles and values of sound leadership practices in their professional life.^3^

In this regard, the Ghana College of Physicians and Surgeons (GCPS), in collaboration with the Ministry of Health (MoH) and the Ghana Health Service (GHS), has established an annual leadership symposium to celebrate examples of excellent and innovative leadership in the health sector in Ghana. This is to inspire the next generation of leaders in the health sector to leverage the achievements of past examples of good leadership to improve the health sector. The overall objective of the symposium is to learn from our past examples of good leadership and use these to influence new thinking and innovation, one of the mandates of the GCPS in providing evidence and information for health sector development.

This year's symposium was held on the 24th of August,2022, at the Auditorium of the Ghana College of Physicians and Surgeons, Accra, Ghana. The theme for the event was “Health Sector Development in Ghana; the Power of Good Leadership”. With a sub-theme of “Leadership in Health and Medical Education”, this year's symposium was held in honour of the legacy of Professor Samuel Ofosu-Amaah, a distinguished Paediatrician and Emeritus Professor of Public Health. As an exemplary leader with a long record of service in the health sector, he has contributed immensely to the education and training of public health experts in Ghana.

One hundred seventy-seven participants attended attended in person, and 60 participated online. Participants comprised those in academia, healthcare providers, health professionals, policymakers, international partners, faculty members and residents of the GCPS, and the family of the distinguished laureate. The framework of the symposium included a background documentary on the laureate's life and achievements, a keynote address, a high-level discussion panel, and an awards ceremony.

The keynote speaker at the symposium was Professor David Ofori-Adjei (Formerly Rector of the Ghana College of Physicians and Surgeons). The panelist comprised Dr Nana Enyimayew (Public Health Specialist), Dr Charlotte Gardiner (Public Health Specialist) and Dr Delanyo Dovlo (Chairman, Faculty of Public Health, GCPS).

Concerning the leadership legacy of Professor Ofosu-Amaah (the laureate), this article reflects on the leadership challenges in the health sector. The lessons learnt from the symposium include the lessons from the personal and professional life of the laureate and the way forward.

## Leadership challenges

### Mentoring

In low-resource countries like Ghana, there is a gap in strategies to build and develop health workers' skills and knowledge to ensure quality service delivery.^4^ Most leaders in this setting, when supervising healthcare workers, are interested in data collection, report writing, and facility appraisals rather than emphasising capacity building and problem-solving.^5^ Mentoring and coaching are ways this gap can be bridged to ensure health system strengthening to achieve UHC. A study in some African countries showed that incorporating mentorship and coaching in our leadership style will result in high quality clinical care, data-driven decision-making, and staff satisfaction while ensuring accountability.^4^

### Teamwork

The coordination and ability to deliver high-quality healthcare depend on teamwork.^6^ Medical errors arise when our healthcare delivery is fragmented in nature.

Accountability, conflict management, decision-making, reflecting on progress and coaching are some of the common challenges facing most teams in the health sector.^7^ Teamwork is needed to ensure a more effective, patient-centred healthcare delivery system. Successes of team-work collaboration need to be incorporated into our medical education and practice across health professions.

### Leadership and governance

Leadership and Governance are critical building blocks of our health system and essential determinants of health system strengthening. A study done in Ethiopia showed that healthcare managers and leaders were incapable of creating a vision, lacked leadership knowledge and skills, and thus could not develop followership.^8^ This affects organisational performance. Bad leadership results in poor governance and management, poor service delivery and overall deterioration of population health. Developing core leadership capabilities is an important phase of leadership development. Exemplary leadership is the solution to challenges confronting organisations today. Leaders ought to lead with care, communicate the vision, develop the capability of their followers, encourage teamwork and ensure accountability.^9^

## Lessons from the symposium as a learning opportunity

### The event

#### Leading an event organisation

This experience taught the public health residents key strategies in leading the planning and organisation of a significant event within the GCPS. Despite the two-month timeline given to the organisers of the event to put things together, the team worked efficiently and collaboratively, utilising all possible networks and means of stakeholder engagement to send out invitations to guests, secure sponsorship and arrange with vendors to provide logistics for the event. Assigned student team leads could effectively allocate duties and tasks in line with meeting the symposium's goals. Additionally, organising a program soliciting funding from development partners requires the event's theme to align with those of prospective funding organisations. This was essentially the case for this symposium, leading to increased patronage and sponsorship from development partners such as the World Health Organisation (WHO). Teamwork was key to achieving healthy working relationships. The effective collaboration between the public health residents, the faculty of public health and the management of the GCPS led to the excellent execution of the event.

The historical recognition of prominent leaders' contributions to the health sector while they are still alive, will go a long way to encourage the younger generation of health professionals to put in their best in their respective roles. Young professionals at the event were motivated by the exemplary life of Professor Ofosu-Amaah. His achievements and leadership skills were worth emulating.

## The personal life of Professor Ofosu-Amaah

### A focus on giving back to the community

The laureate dedicated much of his life to serving and transforming the lives of people in the community. His involvement in establishing the Danfa Comprehensive Rural Health and Family Planning Program was largely aimed at achieving universal health coverage, while the Bamako initiative focused on improving access to medicines in the context of primary health care. These show how ready he was to contribute his quota to the country's health development and beyond.

### Building mentorship of new and upcoming health leaders

The laureate's life reinforces the importance of mentorship in leadership and management. He was an excellent mentor to many prominent individuals who attended the event. This was reflected mainly in comments captured in the video documentary, the discussions by panellists and the words of appreciation in the brochure for the event. Leaders and managers of the health system must set goals, give feedback, and guide the leaders of tomorrow through effective mentorship programmes.

## The professional life of Professor Ofosu-Amaah

### Catalysing change

Professor Ofosu-Amaah's critical decision-making skills were evident in the setting up GCPS amid opposition. He also established the School of Public Health at the University of Ghana, effectively providing a critical mass of leaders at the district and other levels in Ghana over the past two decades. Through networking with stakeholders, he created momentum in public health leadership at operational levels, expanding specialist availability, growth in service delivery and volume of medical training by establishing more medical schools.

### Leadership and governance in public health institutions

Professor Ofosu-Amaah's leadership and governance set him apart from his peers. He worked towards developing and actualising the goals and vision of any organisation he found himself. This was evident in his many leadership roles, such as the Founding Director of the School of Public Health of the University of Ghana, the Inaugural President of the Ghana College of Physicians and Surgeons, the Chair of the Board of Korle-Bu Teaching Hospital and Senior Advisor in health at the UNICEF head-quarters in New York.

### Publication of research findings

The laureate's role in research cannot be overlooked. He generated evidence which served as the basis for the implementation of interventions that have made a significant impact in the health sector. He established a research and training facility for the then Ghana Medical School, which offered community engagement experience to medical students. His many publications are a testament to his knowledge and expertise, which he gladly shared with the scientific community through books, articles, and policy documents.

## Way forward

The gaps in the distribution of specialist medical doctors were critically assessed at the symposium. The findings indicated that their impact on service delivery is not felt across the entire spectrum of catchment populations in all regions of Ghana, despite the cumulative increase in such specialist doctors graduating from the College of Physicians and Surgeons every year. Most of these specialist doctors are concentrated in Accra, Kumasi, Takoradi, Cape coast, Ho and Tamale, widening the gap in access to specialist services in the Ghanaian health sector. This worsens the undesirable outcomes of health service delivery, especially for the people living in semiurban and rural communities. A proposal was made to restructure the posting of specialists trained by the GCPS to ensure equity in the distribution and delivery of specialist healthcare services.

In addition, it was suggested that the School of Public Health of the University of Ghana be named after Professor Samuel Ofosu-Amaah in recognition of his leadership and governance of the school. Furthermore, the GCPS has considered including a leadership and management module in all faculty curricula to provide the required leadership training for all residents, focussing on health sector development. Also, the college plans to establish an “Observatory” on health leadership to focus research on how leadership influences relevant health sector policy issues.
